# Downregulation of semaphorin 3E promotes hallmarks of experimental chronic allergic asthma

**DOI:** 10.18632/oncotarget.22144

**Published:** 2017-10-27

**Authors:** Hesam Movassagh, Lianyu Shan, Jonathan S. Duke-Cohan, Jamila Chakir, Andrew J. Halayko, Latifa Koussih, Abdelilah S. Gounni

**Affiliations:** ^1^ Department of Immunology, Faculty of Health Sciences, College of Medicine, University of Manitoba, Winnipeg, MB, Canada; ^2^ Department of Medical Oncology, Dana-Farber Cancer Institute, Harvard Institutes of Medicine, Boston, MA, USA; ^3^ Centre de recherche de l’Institut universitaire de cardiologie et de pneumologie du Quebec, Universite´ Laval, Quebec City, QC, Canada; ^4^ Department of Physiology & Pathophysiology, Faculty of Health Sciences, College of Medicine, University of Manitoba, Winnipeg, MB, Canada; ^5^ Biology of Breathing Group, Children's Hospital Research Institute of Manitoba, Winnipeg, MB, Canada

**Keywords:** airway hyperresponsiveness, chronic allergic asthma, inflammation, remodeling, semaphorin 3E

## Abstract

Guidance cues such as semaphorins are attractive novel therapeutic targets for allergic disorders. We have previously described an inhibitory effect of semaphorin 3E (Sema3E) on human airway smooth muscle cell function. We have further addressed a canonical role for Sema3E in acute model of allergic asthma *in vivo*. Considering the chronic nature of the disease, the potential implication of Sema3E to alleviate long-lasting deficits should be investigated. Expression of Sema3E in a chronic model of allergic asthma was assessed after exposure to house dust mite (HDM) as a clinically relevant allergen. Chronic features of allergic asthma including airway hyper-responsiveness (AHR), inflammation, and remodeling were studied in Sema3E-deficient mice. Additionally, the effect of exogenous Sema3E treatment was evaluated in prophylactic and therapeutic experimental models. We have demonstrated that expression of Sema3E is robustly suppressed in the airways upon chronic HDM exposure. Chronic allergic airway disease was significantly augmented in Sema3E-deficient mouse model which was associated with an increased AHR, remodeling, and Th2/Th17 inflammation. Intranasal Sema3E administration restored chronic deficits of allergic asthma in mice. Data from this study unveil a key regulatory role of Sema3E in chronic course of asthma via orchestration of impaired inflammatory and remodeling responses.

## INTRODUCTION

Asthma is one of the most common chronic diseases which affects approximately 300 million people worldwide. Despite remarkable advances in asthma treatment, optimal control of the disease remains unachieved particularly among those of inhaled corticosteroid users suffering chronic symptoms [1]. Major impacts on quality of life and economic burden necessitate a better understanding about the determinants of uncontrolled asthma [2, 3]. Chronic allergic asthma is characterized by persistent airway inflammation, hyperresponsiveness (AHR) and structural changes in the lungs collectively known as airway remodeling [4, 5]. AHR is an exaggerated airway narrowing caused by nonspecific irritants or agonists associated with increased lung infiltration by inflammatory cells [6]. The persistent AHR in patients refractory to steroid therapy is associated with airflow limitation or remodeled airway leading to a change in mechanical properties [7]. The precise mechanisms underlying pathological features of chronic asthma are far from resolved.

Sensitization to indoor aeroallergens occurred upon chronic exposure is an essential determinant of asthma control. Chronic encounter with house dust mite (HDM), as a common indoor allergen, causes early airway damage during the childhood results in irreversible destruction of the airways via bronchial remodeling [8, 9]. Current asthma therapies fail to effectively reduce bronchial remodeling and inflammation particularly in uncontrolled severe asthmatics.

Semaphorins are guidance cues ubiquitously expressed in different organs and play diverse regulatory roles in various cellular functions. For instance, we have previously addressed the inhibitory effect of semaphorin 3E (Sema3E) on human airway smooth muscle (ASM) in the airways of severe asthmatic patients cell proliferation and migration as a characteristic feature of airway remodeling [10]. Furthermore, we have recently demonstrated a significant reduction of Sema3E expression in the airways of severe asthmatic patients [11]. Our recent studies on an acute model of the disease indicate that Sema3E could play an essential role in allergic asthma by regulating the function of neutrophils and dendritic cells [12–14]. However, the potential contribution of Sema3E in chronic features of asthma has remained to be addressed. In this study, we have hypothesized that Sema3E negatively regulates chronic features of allergic asthma induced by consecutive HDM exposure. In addition, we aim to assess translational implication of Sema3E treatment in both therapeutic and prophylactic contexts.

We reveal that chronic HDM exposure downregulates Sema3E expression in murine airways. Sema3E-deficient mice developed a spontaneous augmented AHR, inflammation, collagen deposition, and mucus overproduction. Intranasal treatment with exogenous Sema3E alleviated chronic features of allergic asthma and restored airway homeostasis via regulation of mediators involved in asthma pathology.

## RESULTS

### Sema3E expression is suppressed in the airways by chronic HDM exposure in mice

**Figure 1:**
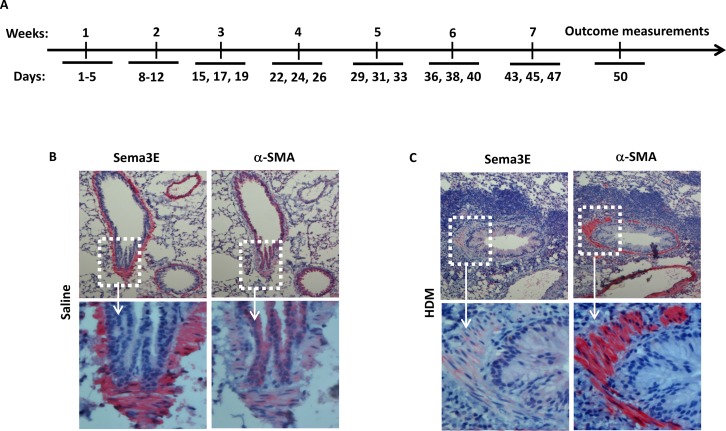
Sema3E expression is suppressed upon chronic HDM exposure A chronic model of allergic asthma was established by intranasal administration of HDM for 7 weeks **(A)**. Mouse Sema3E immunoreactivity was investigated by performing in lung tissue section obtained normal **(B)** and chronic asthmatic **(C)** mice. Sema3E expression was visualized after incubation of tissue sections with anti-Sema3E specific antibody, Fast-red development and counterstaining by 100X (upper panels) and 400X (lower panels) magnifications. α-SMA staining was also performed on the tissue sections obtained from the same mice (B-C). (n=4 mice per group).

We previously reported a decreased surface expression of Sema3E receptor, PlexinD1, on human ASM cells from asthmatic patients [10]. However, expression of Sema3E has not been investigated in mouse chronically exposed to HDM. We examined the expression of Sema3E in lung tissue sections obtained from the mice exposed to either a chronic intranasal HDM or saline regimen as depicted in Figure 1A. By performing immunohistochemistry we revealed that Sema3E is highly expressed on the bronchial epithelial cells of the normal saline-treated mice (Figure 1B). Interestingly, chronic intranasal sensitization and challenge with HDM for seven weeks induced a remarkable decrease in Sema3E immuno-reactivity at the epithelial layer and myofibroblasts (Figure 1C). This downregulation of Sema3E was concomitant with increased ASM mass, evident by the overexpression of alpha smooth muscle actin (α-SMA), and recruitment of inflammatory leukocytes to the airways (Figure 1C).

### Sema3E deficiency augments AHR upon HDM chronic exposure

**Figure 2:**
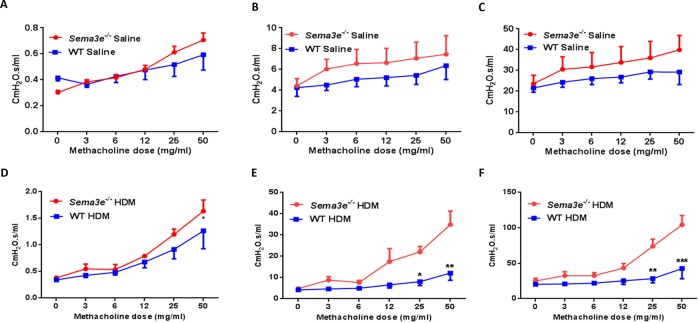
Sema3E deficient mice show enhanced AHR upon chronic HDM exposure *Sema3e^−/−^* mice or WT littermates exposed to HDM for 7 weeks underwent tracheotomy accompanied by methacholine challenge to measure AHR parameters including airway resistance **(A** and **D)**, tissue resistance **(B** and **E)**, and tissue elastance **(C** and **F)**. The data represent two independent experiments on age and sex-matched mice. (n = 5-6 per group, ^*^P<0.05, ^**^P<0.01. and ^***^P<0.001).

As AHR is a hallmark clinical manifestation of allergic asthma [15], we first assessed the effects of Sema3E gene deletion on basal and HDM-induced AHR by studying lung function parameters. *Sema3e^−/−^* and WT mice underwent anaesthesia and then tracheotomy followed by intra-tracheal instillation with increasing concentrations of methacholine. *Sema3e^−/−^* mice had a slightly higher airway resistance (Figure 2A), tissue resistance (Figure 2B) and tissue elastance (Figure 2C) at the baseline. AHR parameters were significantly heightened in *Sema3e^−/−^* mice compared with WT littermates upon chronic HDM sensitization and challenge (Figure 2D-2F). Therefore, our data suggest that Sema3E is involved in regulation of HDM-induced AHR.

### Mucus overproduction and collagen deposition is aggravated in Sema3E-deficient mice

**Figure 3:**
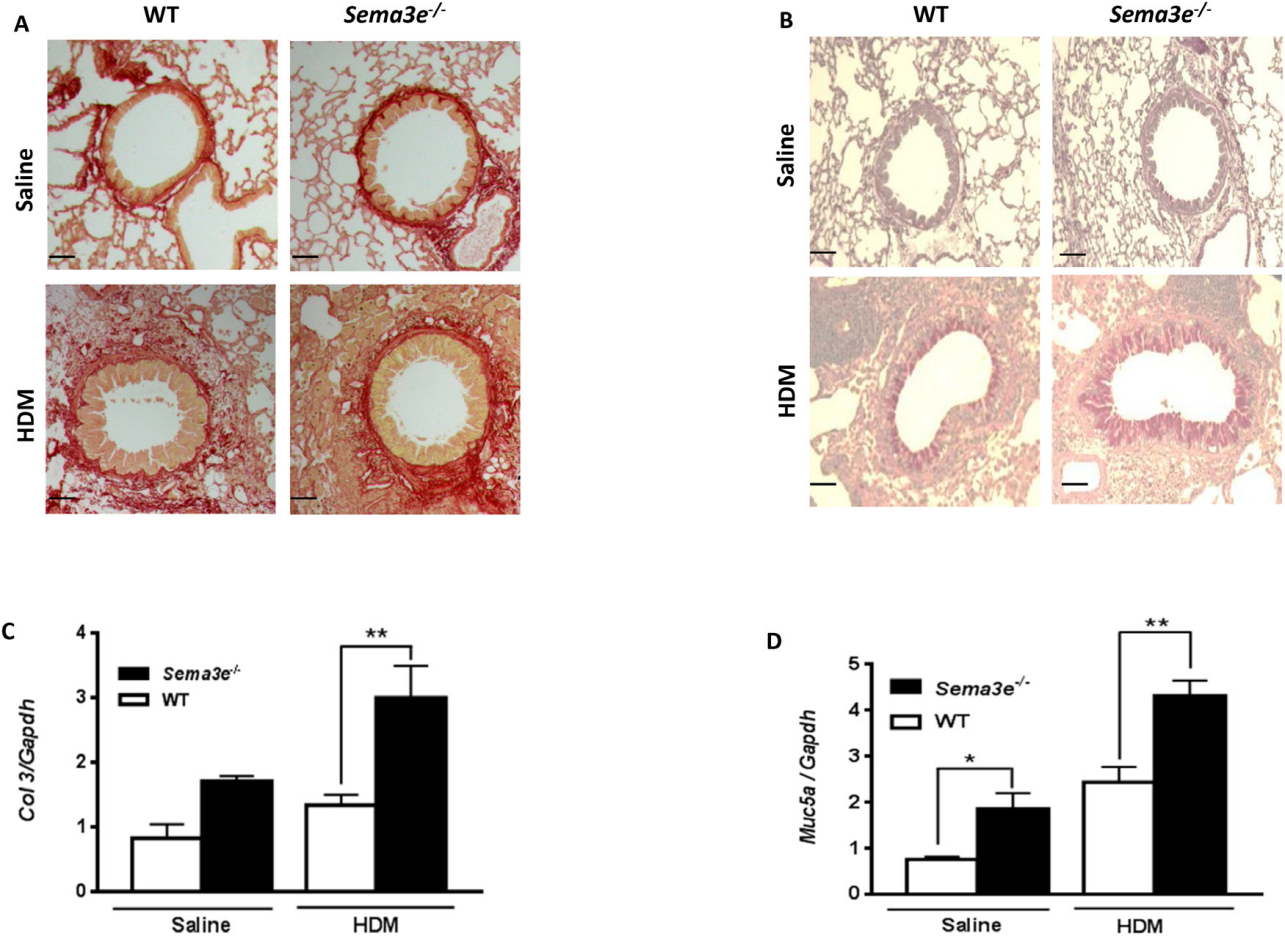
Collagen deposition and mucus overproduction is elevated in Sema3E deficient mice Lung tissue sections from saline- or HDM-exposed *Sema3e^−/−^* and WT mice were stained with Sirius red **(A)** and PAS **(B)** to determine collagen deposition and mucus hypersecretion, respectively (Scale bars: 100μm). Expression of remodeling genes, *Col3* **(C)** and *Muc5ac* **(D)**, in the lungs of *Sema3e^−/−^* and WT mice at the baseline or upon HDM challenge was studied by quantitative real-time PCR using specific primers (n= 5-6 per group, ^*^P<0.05 and ^**^P<0.01).

We examined the effect of Sema3E deficiency on structural alterations in the airways. To determine whether Sema3E plays a role in the development of airway remodeling, we first evaluated peribronchial fibrosis, characterized by deposition of collagen [16], in either HDM- or saline-exposed *Sema3e^−/−^* versus WT mice. Sirius red staining on lung tissue sections demonstrated that exposure to HDM enhanced collagen deposition in *Sema3e^−/−^* mice more significantly than those of WT counterparts (Figure 3A). In addition, it has been previously shown that airway obstruction in chronic asthma is partly mediated by mucus plugging in which mucus is overproduced by goblet cells [17, 18]. Therefore, we assessed the mucin level by PAS staining on lung tissue sections obtained from *Sema3e^−/−^* and WT mice. Positively stained for mucin with PAS was significantly increased upon HDM chronic exposure in the airway epithelium of *Sema3e^−/−^* mice compared to the WT littermates (Figure 3B). Increased collagen deposition and mucus overproduction in *Sema3e^−/−^* mice was further confirmed at mRNA level by performing qPCR to study expression of *Col3* and *Muc5ac*, respectively (Figure 3C-3D). Altogether, deficiency of Sema3E increased collagen deposition and goblet cell metaplasia as the characteristic features of airway remodeling.

### Sema3E deficiency enhances chronic allergic airway inflammation

**Figure 4:**
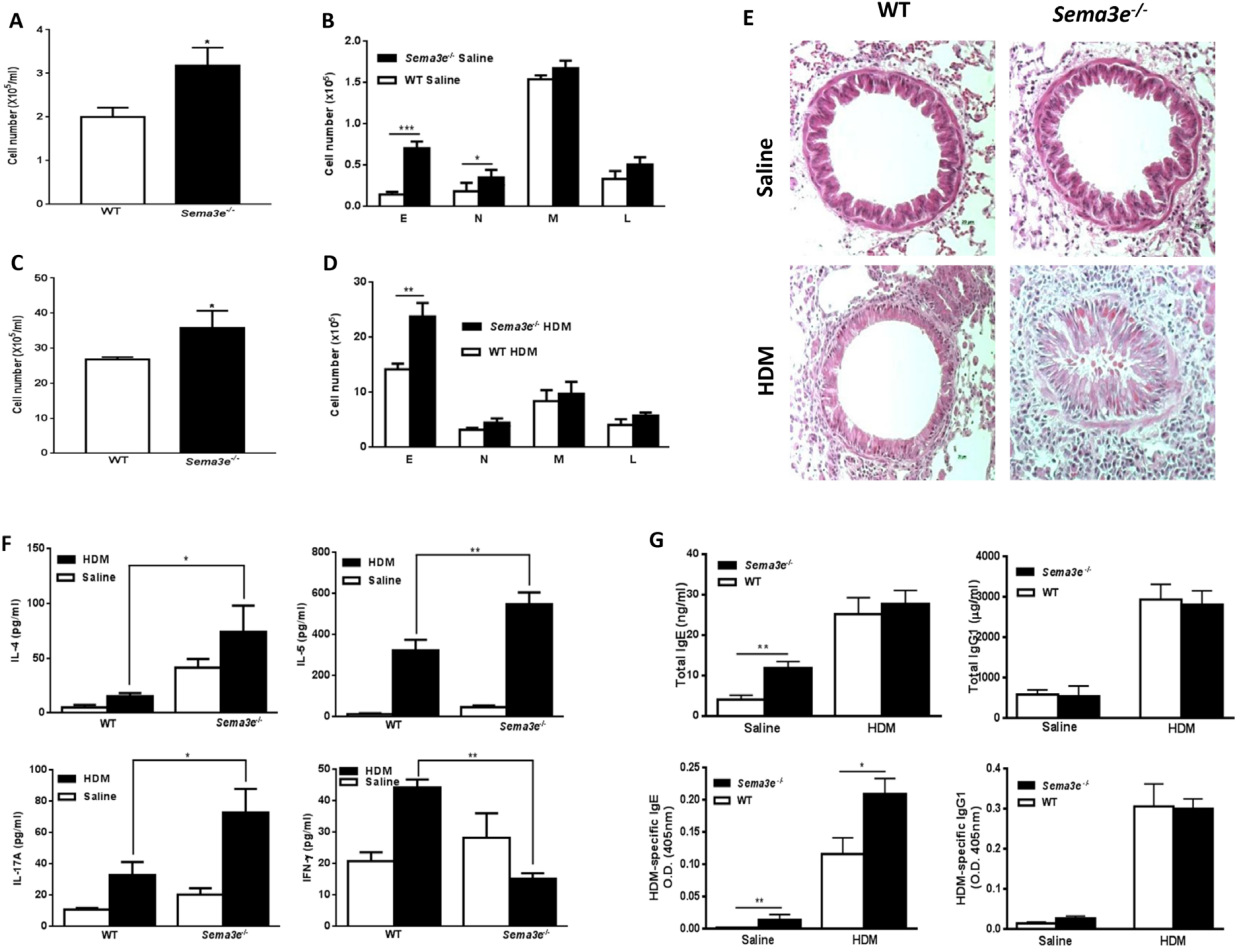
Basal and HDM-induced chronic airway inflammation is increased in Sema3E deficient mice Total and differential cell count was performed on BAL fluid from either *Sema3e^−/−^* or WT mice after saline **(A-B)** or HDM **(C-D)** exposure. Airway inflammation was studied by performing H&E on lung tissue sections **(E)**. Airway levels of IL-4, IL-5, IL-17A and IFN-γ were measured upon either HDM or saline intranasal exposure by ELISA in BAL fluid obtained from *Sema3e^−/−^* or WT mice **(F)**. Serum level of total and HDM specific IgE and IgG1 of exposed saline or HDM sema3-e- and WT mice were determined by ELISA **(G)**. E: eosinophil, N: neutrophil, M: macrophage, L: lymphocyte. (Scale bars: 100μm, n = 5-6 per group, ^*^P<0.05, ^**^P<0.01 and ^***^P<0.001).

We evaluated the role of Sema3E in recruitment of inflammatory cells into the airways by analyzing cytospins obtained from BAL fluid. The total number of inflammatory cells and number of eosinophils and neutrophils were significantly higher in BAL fluid from *Sema3e^−/−^* mice than WT littermates at the baseline (Figure 4A-4B). HDM exposure induced an eosinophilic inflammation which was more pronounced in the absence of Sema3E (Figure 4C-4D). H&E-stained lung sections further demonstrated a remarkable increase in magnitude of peribronchial inflammatory infiltrates of *Sema3e^−/−^* mice compared to the WT controls (Figure 4E).

We also assessed the level of cytokines involved in pathogenesis of allergic asthma in BAL fluid from WT and *Sema3e^−/−^* mice. The concentration of Th2 (IL-4 and IL-5) and Th17 (IL-17A) cytokines were significantly increased in BAL fluid from HDM-exposed *Sema3e^−/−^* mice compared to WT control group (Figure 4F). In contrast, HDM exposure significantly reduced the level of Th1 cytokine, IFN-γ, in the absence of Sema3E (Figure 4F). Increased Th2/Th17-skewed cytokine response in *Sema3e^−/−^* mice after HDM chronic exposure was further confirmed by performing intracellular staining of IL-4, IL-17A and IFN-γ in CD4^+^ T cells from the lung draining MLN (Supplementary Figure 1). Considering the importance of IgE and IgG1 in allergic asthma [19, 20] we measured the levels of total and HDM-specific forms of these antibodies in the sera obtained from *Sema3e^−/−^* and WT mice. As shown in Figure 4G, genetic deletion of Sema3E enhanced total IgE level in naïve; but not in HDM-exposed mice. However, HDM-specific IgE level was elevated in both saline and HDM-exposed *Sema3e^−/−^* mice compared to the WT littermates. Total or HDM-specific level of IgG1 was not significantly different between *Sema3e^−/−^* and WT mice at the baseline nor after chronic HDM challenge. Thus, deletion of Sema3E heightens airway inflammatory cellular infiltrate, induces a Th2/Th17-deviated response and increases IgE synthesis.

### Intranasal administration of Sema3E inhibits the AHR, remodeling and airway inflammation

**Figure 5:**
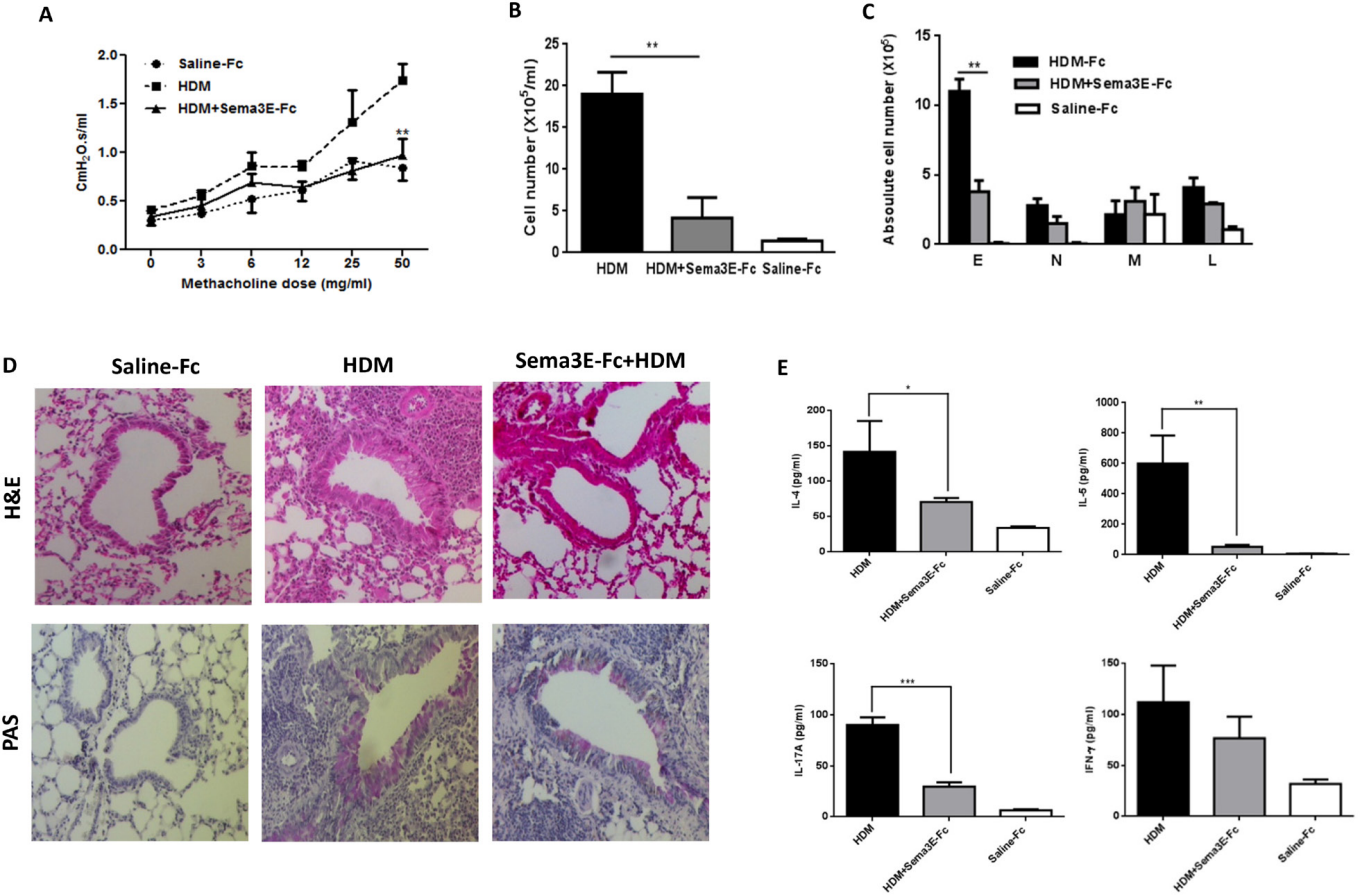
Intranasal administration of exogenous Sema3E prevents development of HDM-induced chronic inflammation Intranasal administration of Sema3E prevents HDM-induced airway resistance **(A)**. Sema3E also reduced recruitment of total inflammatory cells **(B)**, specifically eosinophils **(C)**. Inhibitory effect of Sema3E treatment on pulmonary inflammation and mucus hyper-secretion was addressed by performing H&E and PAS staining, respectively **(D)**. HDM-induced secretion of IL-4, IL-5, and IL-17A into the airways was significantly abolished by Sema3E administration **(E)**. (n=4, ^*^P<0.05, ^**^P<0.01 and ^***^P<0.001).

In order to address the potential protective effect of Sema3E in chronic allergic airway disease, we administered exogenous recombinant Sema3E 1h before each HDM exposure. Then, lung function parameters in response to an increasing dose of nebulized methacholine were measured. HDM-induced conducting airways resistance (Figure 5A), tissue resistance and tissue elastance (Supplementary Figure 2A-2B) was significantly reduced in mice receiving intranasal Sema3E prior to HDM exposure.

Administration of recombinant Sema3E significantly reduced the level of total inflammatory cells (Figure 5B) and eosinophils (Figure 5C) evoked by HDM in the airways. There was no significant effect of Sema3E on HDM-induced recruitment of neutrophils, lymphocyte or macrophages (Figure 5C). Staining with H&E further revealed that Sema3E treatment inhibited HDM-induced perivascular and peribronchial accumulation of inflammatory cells in the lungs (Figure 5D). Th2/Th17 cytokine response (Figure 5E) as well as total and HDM-specific forms of both IgE (Supplementary Figure 3A-3B) and IgG1 synthesis (Supplementary Figure 3C-3D) induced by HDM chronic exposure was abolished upon Sema3E treatment. However, Sema3E did not significantly change the levels of IFN-γ (Figure 5E). Furthermore, Sema3E treatment prevented HDM-induced mucus overproduction (Figure 5D).

### Treatment with Sema3E post chronic HDM challenge reverses AHR, remodeling, and airway inflammation

**Figure 6:**
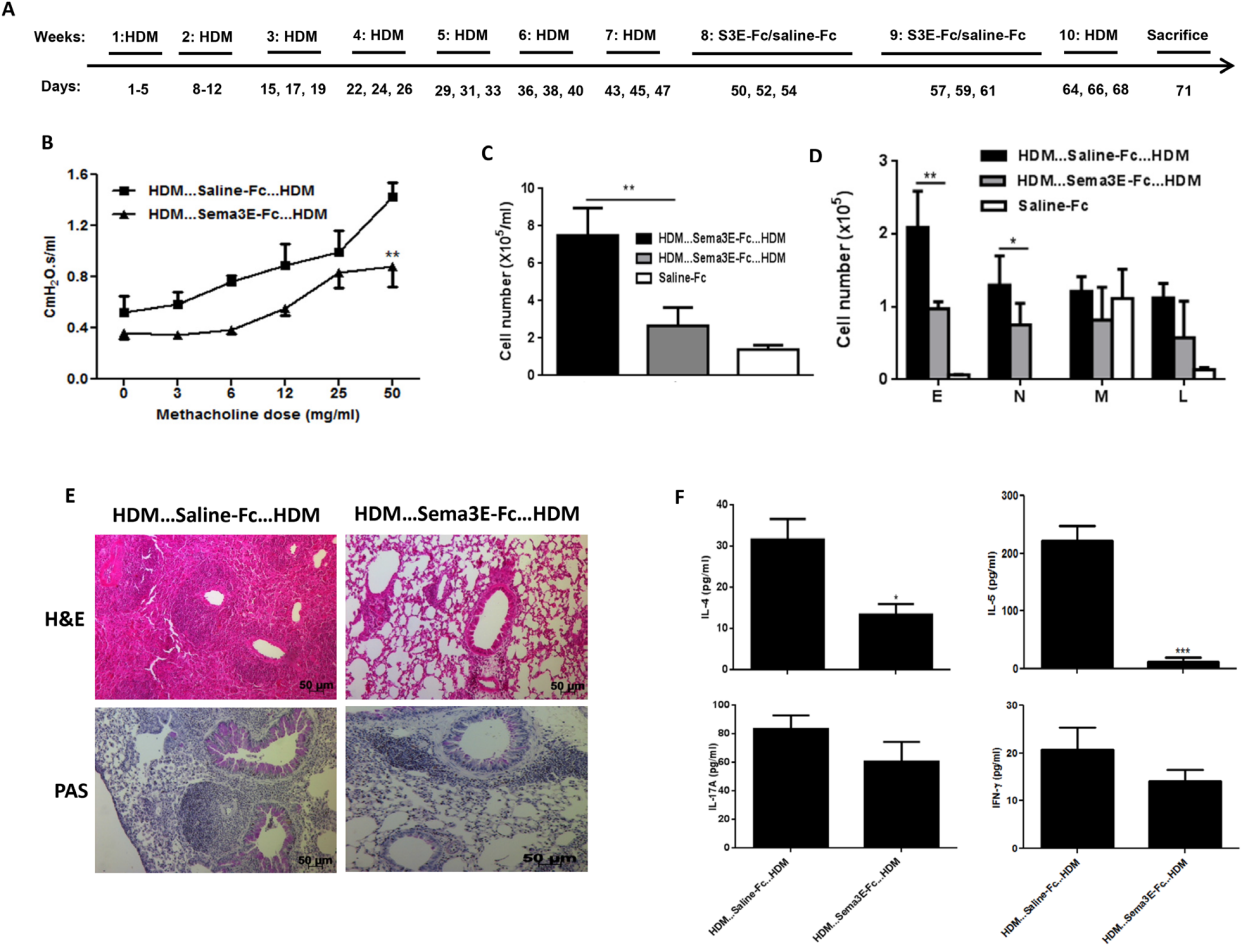
Intranasal Sema3E treatment attenuates chronic allergic asthma deficits upon HDM re-exposure Mice were exposed with HDM for 7 weeks then treated with Sema3-Fc or Fc-Ig control then exposed to HDM for one more week **(A)** Intranasal administration of Sema3E protects mice against HDM-induced airway resistance after re-exposure with HDM **(B)**. Sema3E treatment also reduced total **(C)** and granulocytic **(D)** recruitment of inflammatory cells into the airways. Pulmonary inflammation and mucus hyper-secretion were decreased upon Sema3E treatment **(E)**. Decreased secretion of Th2 and Th17 cytokines by Sema3E was sustained upon HDM re-exposure. **(F)** E: eosinophil, N: neutrophil, M: macrophage, L: lymphocyte. (n=4, ^*^P<0.05, P<0.01 and ^***^P<0.001).

We established chronic asthma model by intranasal HDM exposure for 5 consecutive weeks and then treated mice with either Sema3E or saline for another 2 weeks then re-challenged with HDM for one week (Figure 6A). This protocol will allow us to evaluate whether the therapeutic effect of Sema3E is long lasting. Our outcome measurements demonstrated that intranasal treatment of chronic asthmatic mice with recombinant Sema3E protects them from developing exaggerated airway resistance (Figure 6B), tissue resistance (Supplementary Figure 4A), and tissue elastance (Supplementary Figure 4B). The total number of airway inflammatory cells (Figure 6C) as well as eosinophils and neutrophils (Figure 6D) was significantly decreased in Sema3E-treated group. The therapeutic effect of Sema3E upon HDM re-exposure was associated with reduced Th2/Th17 airway inflammation (Figure 6E-6F) and mucin level (Figure 6E). Finally, Sema3E reduced both total and HDM-specific IgE (Supplementary Figure 5A-5B) and total IgG1 (Supplementary Figure 5C) levels; while did not significantly alter HDM-specific IgG1 synthesis (Supplementary Figure 5D). Collectively, these data suggest that Sema3E could play an essential inhibitory role in reversing pathological features of chronic allergic asthma.

## DISCUSSION

In our recent studies, we have uncovered that Sema3E plays a crucial regulatory role in an acute model of allergic asthma. Acute features of asthma were enhanced in *Sema3e^−/−^* mice [13]. However, the chronic nature of the disease necessitates investigating the role of Sema3E in a chronic model of the disease. It enables us to particularly address whether the regulatory role of Sema3E sustains over the time and decipher its role in a prolonged AHR.

In the present study, we demonstrated that chronic exposure to HDM markedly reduces Sema3E immunoreactivity in mouse airways suggestive of a potential protective role for this mediator against chronic deficits of allergic asthma. We also revealed that in chronic mouse model of allergic asthma genetic abrogation of Sema3E exaggerates the development of AHR, remodeling and inflammation. Genetic deletion of Sema3E could be analogous to asthmatic conditions in which expression of Sema3E has been pathologically suppressed by an unknown mechanism. Of great therapeutic importance, administration of extrinsic recombinant Sema3E, during or after development of the disease, alleviates chronic asthma facets. Therefore, we propose that replenishment of Sema3E could be a protective strategy during chronic HDM challenge which is associated with the downregulation of Sema3E.

Remarkable reduction of Sema3E in the chronic model of allergic asthma is similar to our previous findings on the acute model of the disease [12] and also severe human patients [11]. However, it is not still clear that whether allergen exposure primarily induces downregulation of Sema3E; genetic defect of Sema3E leads to a heighten asthma pathology; or a combination of both possibilities are involved. It deserves further developmental studies to determine whether Sema3E downregulation is a cause or consequence of allergen exposure.

AHR is a key clinical feature of allergic asthma induced by chronic type 2 inflammation [21]. Therefore, reduction of AHR via balancing type 2-biased allergic inflammation by Sema3E could be an underlying mechanism of Sema3E action in asthma. In addition, inflammation-independent AHR, mediated by structural cells [22], could be reversed by direct effect of Sema3E on these cells. For instance, Sema3E downregulation could promote ASM cell hyperplasia or contraction that eventually leads to enhanced AHR parameters observed in *Sema3e^−/−^*mice and decreased upon Sema3E treatment.

Increased lung fibrosis evident by increased deposition of collagen in the absence of Sema3E which was reversed upon Sem3E treatment could be attributed to an unknown mechanism in which Sema3E functions as an anti-fibrotic mediator probably via reduction of migration and proliferation of fibroblasts as the main source of collagen production [23]. It could be similar to our previous report where we demonstrated the inhibitory effect of Sema3E on human ASM cells [10]. Enhanced pulmonary fibrosis leads to an increased lung stiffness which could be responsible for the increase in AHR observed in *Sema3e^−/−^*mice. The anti-fibrotic effect of Sema3E could be investigated in models of idiopathic pulmonary fibrosis in which interstitial fibrosis and collagen deposition causes lung damage and respiratory failure [24]. HDM-mediated mucus overproduction which is another crucial facet of structural changes in the airways was enhanced in the absence of Sema3E and reduced by its intranasal administration. It could be speculated that Sema3E might directly downregulate expression of genes responsible for mucus production in goblet cells, e.g. *Muc5ac*, or indirectly reduce expression of mediators, e.g. IL-13, which promote goblet cell hyperplasia [25, 26].

Increased levels of IL-5 and IL-17A in *Sema3e^−/−^* and also their decrease upon Sema3E treatments could explain, at least in part, the mechanism by which Sema3E plays a crucial role in regulation of eosinophil and neutrophil recruitment to the airways [27, 28]. Secretion of both IL-5 and IL-17A is elevated in allergic asthma which is associated with eosinophilic and neutrophilic endotypes, respectively [27, 29]. Considering their biological functions, these cytokines are potential therapeutic targets in allergic asthma [27, 30, 31]. Our study suggests that Sema3E could be considered as a novel treatment for chronic allergic asthma via regulation of both Th2, e.g. IL-4 and IL-5, and Th17, e.g. IL-17A, associated cytokines. Higher levels of HDM-specific IgE in the sera from naïve and HDM-challenged *Sema3e^−/−^* mice as well as reduction of IgE secretion upon exogenous Sema3E treatment suggest a negative unknown regulatory role for Sema3E in IgE class-switching as a cardinal feature of B cell development attributed to IL-4 response [32–34].

In order to completely understand the mechanism underlying therapeutic effect of Sema3E on HDM chronic model of asthma, a detailed investigation of is PlexinD1 expression and function is required. PlexinD1 has been shown to interact with semaphorin 4A which is involved in development of type 2 inflammation [35]. Furthermore, Sema3E signaling is mediated by co-receptors such as neuropilin 1 (Nrp1) and vascular endothelial growth factor receptor 2 (VEGFR2) in a context-dependent manner [36–38]. Therefore, the potential role of these molecules in the HDM model should be studied upon Sema3E administration.

In summary, reduction of Sema3E expression in chronic mouse model of allergic asthma suggests a potential role of this mediator in this disease. Enhanced AHR, remodeling, and inflammation in Sema3E-deficinet mice as well as reversion of these pathological features upon Sema3E treatment provide a novel biological therapeutic approach for chronic allergic asthma in the future.

## MATERIALS AND METHODS

### Animals

*Sema3e^−/−^* mouse (129 P2 strain) was kindly gifted by Dr. F. Mann (Developmental Biology Institute of Marseille Luminy, Université de la Méditerranée, Marseille, France) [37]. Wild-type (WT) 129 P2 strain was used as the littermate controls. In addition, female 6-8 week old Balb/c mice purchased from the Central Animal Care Services (CACS) University of Manitoba were used in the treatment models. All of the mice were maintained under specific pathogen-free conditions and used according to guidelines stipulated by the Canadian Council for Animal Care.

### House dust mite exposure model

Lyophilized HDM protein extract was obtained from Greer Laboratories (Lenoir, NC). Working concentration (25 μg per mouse) was freshly prepared. Chronic model of the disease was established via intranasal administration under gaseous anesthesia for 5 days per week during the first two weeks. HDM was continuously administered via intranasal route every two days for the next 5 weeks, with 2 days interval between those weeks (Figure 1A) [39]. In some experiments, recombinant mouse Sema3E-Fc [40] or saline-Fc (10μg/kg in PBS) was intranasally administered 1h prior to HDM exposure for 7 weeks in Balb/c mice. Otherwise, HDM exposure was stopped at the end of seven week followed by Sema3E-Fc treatment for the next two weeks and then HDM re-exposure every two days for one more week.

### Immunohistochemistry

Formalin-fixed lung tissues were paraffin embedded, and 5-μm-thick sections were prepared, dewaxed in xylene, and rehydrated through graded concentrations of alcohol to water. Then, antigen retrieval was performed in boiling sodium citrate buffer for 10 min. Sections were incubated with blocking buffer for 1h at room temperature, followed by overnight incubation with either anti-mouse Sema3E antibody (R&D Systems, Minneapolis, MN) or isotype control IgG (Jackson ImmunoResearch Laboratories, West Grove, Pa) at 4°C. Slides were then washed twice with TBS followed by incubation for 1h at room temperature with biotin-conjugated secondary antibody. After extensive washing with TBS, slides were incubated with streptavidin-alkaline phosphatase for 30 min at room temperature. Development was performed using Fast Red (Sigma-Aldrich, Oakville, ON, Canada) and counterstained with modified Mayer's hematoxylin (Fisher Scientific, Fair Lawn, NJ). Finally, slides were mounted and visualized using AxioVision software (Carl Zeiss, Inc, Thornwood, NY).

### Airway hyperresponsiveness measurements

AHR parameters including airway resistance, tissue resistance and tissue elastance were measured using FlexiVent small animal ventilator system (SCIREQ, Montreal, QC, Canada). Briefly, HDM or saline sensitized and challenged mice underwent tracheotomy. Then, an increasing gradient of methacholine dose (0, 3, 6, 12, 25 and 50 mg/ml) was administered intratracheally with 5 min interval between the doses and lung function measurements were performed.

### Lung histology

Lower left lobe of the lung was dissected, inflated and fixed in formalin overnight followed by embedding in paraffin. Basal and HDM-induced airway inflammation, mucus overproduction, and collagen deposition in lung tissue sections was studied by performing H&E, Periodic Acid-Schiff (PAS) and Sirius Red staining, respectively.

### RNA isolation and real-time PCR

The right middle lobe of the lungs was homogenized in Trizol® (Life Technologies, Burlington, ON) followed by subsequent RNA isolation and cDNA synthesis, according to the manufacturer's instructions. Then, expression of *Col3* and *Muc5ac* was investigated using specific murine primers.

### Collection of bronchoalveolar lavage fluid

Bronchoalveolar lavage (BAL) fluid was collected from tracheally cannulated mice with two instillations of 1 ml of sterile saline containing 0.1 mM EDTA. After RBC lysis, total BAL fluid cells were counted. Cytospins prepared from the BAL were fixed and stained to perform differential inflammatory cell count. BAL fluid supernatants were stored at -80°C for assessing the cytokine levels.

### Measurement of cytokines and immunoglobulins

The level of IL-4, IL-5, IL-17A, and IFN-γ in BAL fluid was measured by ELISA according to the manufacturer's instructions. All cytokine ELISA kits were from BioLegend (San Diego, CA). Total and HDM-specific forms of IgE and IgG1 levels were quantified using commercial ELISA kits in serum (Southern Biotech Birmingham, Al) as described previously [41].

### Flow cytometry

Mediastinal lymph nodes (MLN) were collected and single cell suspension was prepared. The cells were re-suspended in DMEM and incubated with a freshly prepared cocktail containing 50 ng/ml PMA, 500 ng/ml ionomycin and 10 μg/ml brefeldin A (Sigma-Aldrich, Oakville, ON) for 4h at 37°C and 5% CO_2_. Extracellular staining was performed using anti-mouse CD3 e-Fluor® 450 (Clone: 17A2) and CD4-FITC (Clone: RM4-5) both from eBioscience. After fixation with paraformaldehyde for 15 min at 4°C, cells were permeabilized with 0.1% saponin. Finally, intracellular staining was performed using anti-mouse IL-4-PE (Clone: 11B11), IL-17A-PE (Clone: eBio17B7), and IFN-γ-PE (Clone: XMG1.2). Samples were acquired on a FACSCantoII and analyzed using FlowJo software.

### Statistics

GraphPad Prism 5.0 software was used for the statistical analysis. Data were analyzed by unpaired *t*-test, one-way or two-way ANOVA, followed by the Bonferroni's multiple comparisons post-hoc test. Differences were considered to be statistically significant at ^*^P≤0.05, ^**^P≤0.01 and ^***^P≤0.001.

## SUPPLEMENTARY MATERIALS FIGURES



## Abbreviations

AHR: airway hyperresponsiveness
ASM: airway smooth muscle
BAL: bronchoalveolar lavage
HDM: house dust mite
MLN: mediastinal lymph nodes
Nrp1: neuropilin 1
PAS: Periodic acid-Schiff
Sema: semaphorin
VEGFR2: vascular endothelial growth factor 2
WT: wild type.
