# Supplementary material for: Downregulation of semaphorin 3E promotes hallmarks of experimental chronic allergic asthma
Source: Oncotarget. 2017 Oct 27;8(58):98953–63. doi: 10.18632/oncotarget.22144 (PMC5716780; doi:10.18632/oncotarget.22144)
Supplement: Supplementary file 1 [file oncotarget-08-98953-s001.pdf]

Downregulation of semaphorin 3E promotes hallmarks of experimental chronic allergic asthma

SUPPLEMENTARY MATERIALS

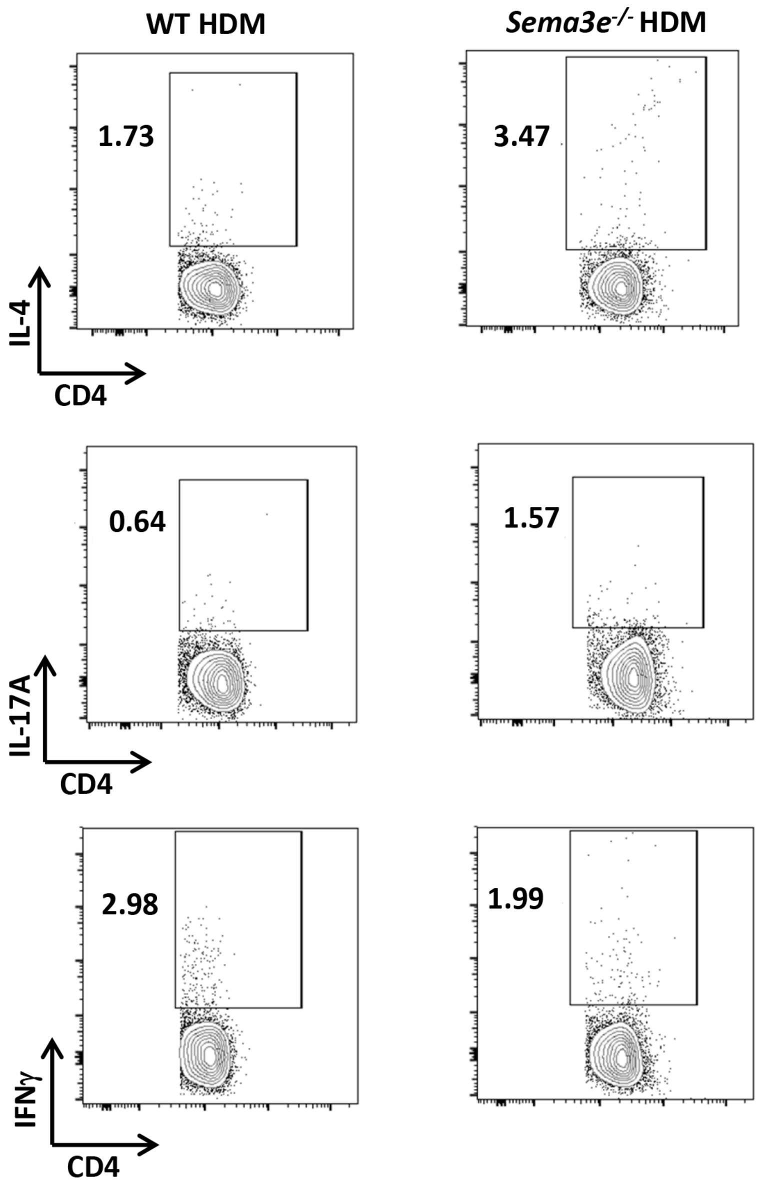

Supplementary Figure 1: Sema3E deficiency leads to Th2/Th17-skewed cytokine response upon HDM challenge in mice. Intracellular production of IL-4, IL-17A, and IFN-γ was investigated in $CD4^+$ MLN cells obtained from *Sema3e*$^{-/-}$ or WT mice after chronic exposure to HDM by flow cytometry using specific antibodies *ex vivo* (n = 5-6 mice per group).

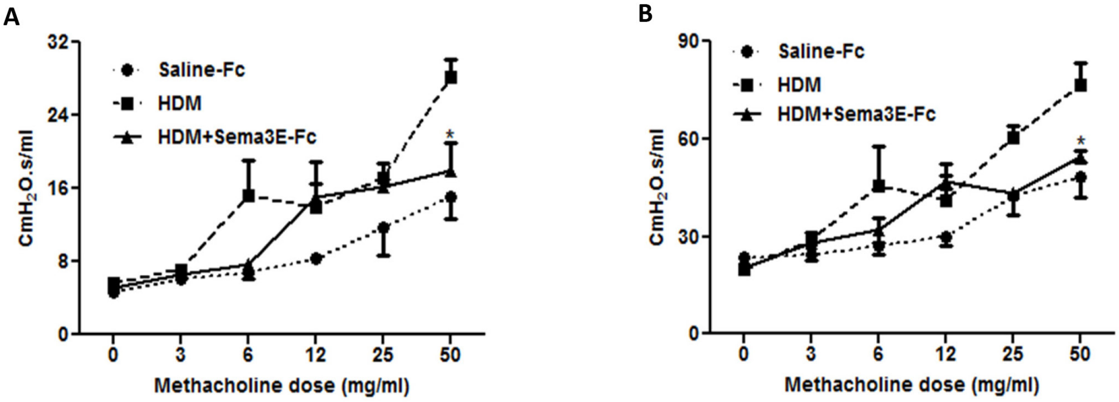

Supplementary Figure 2: Sema3E treatment prevents HDM-induced pulmonary resistance and elastance. Protective effect of intranasal Sema3E administration on lung tissue resistance **(A)** and elastance **(B)** was evaluated in chronically HDM-challenged mice by a FlexiVent system. (n=4 mice per group, **P<0.05)*.

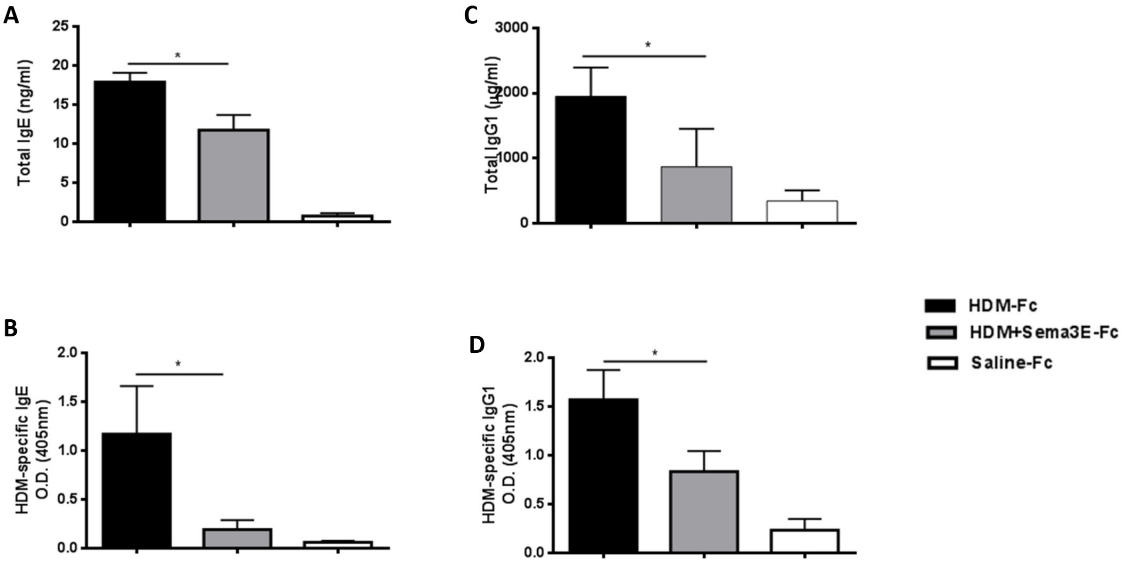

Supplementary Figure 3: Sema3E treatment negatively regulates HDM-induced IgE and IgG1 synthesis. Total **(A)** and HDM-specific IgE **(B)** as well as IgG1 **(C-D)** were measured in the serum samples obtained from the mice treated with Sema3E before HDM challenge by ELISA (n=4 mice per group, $*P<0.05$).

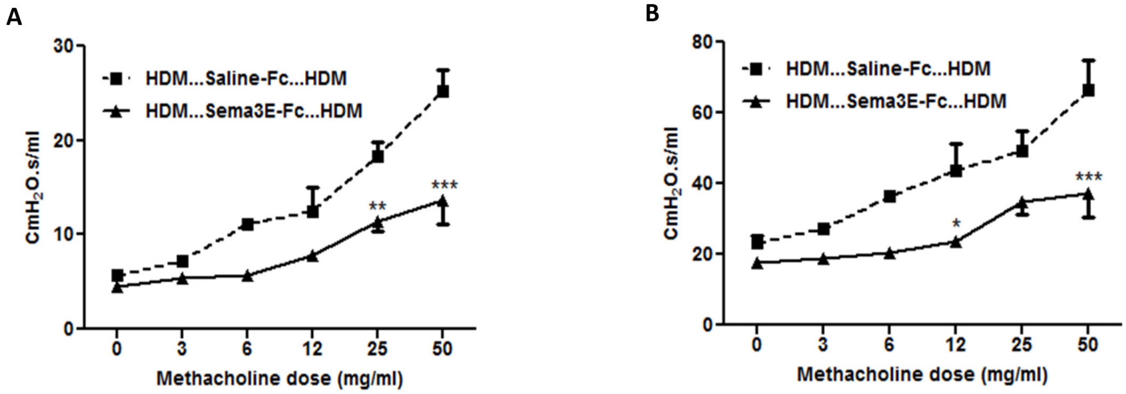

Supplementary Figure 4: Sema3E-mediated reduction of pulmonary resistance and elastance sustains upon HDM re-exposure. The effect of intranasal treatment with Sema3E on lung tissue resistance **(A)** and elastance **(B)** was evaluated using a FlexiVent system in an established chronic model after HDM re-exposure. (n=4 mice per group, *$P<0.05$, **$P<0.01$, and ***$P<0.001$).

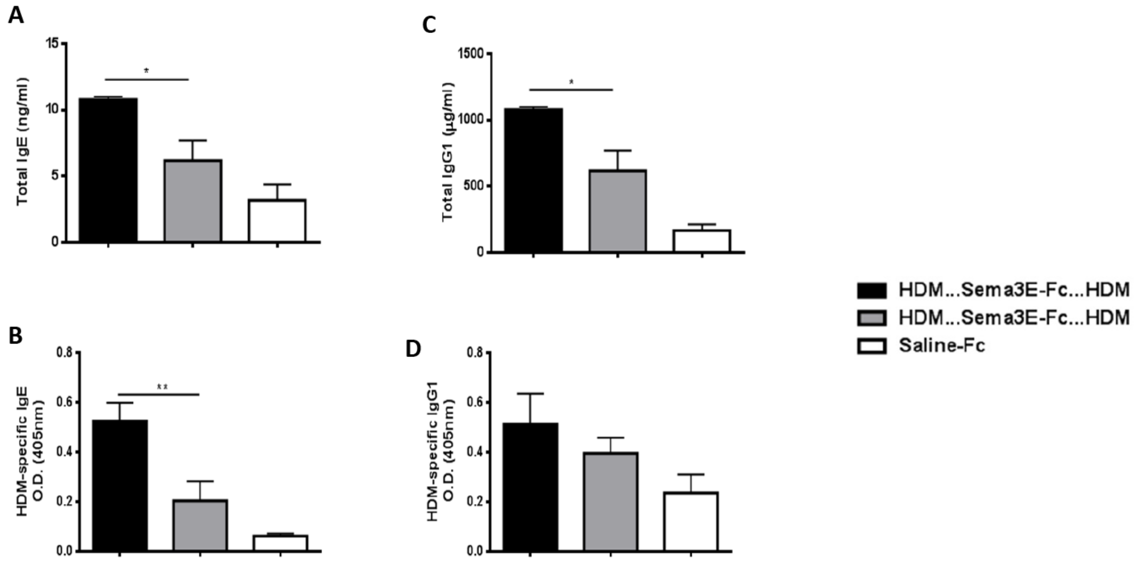

Supplementary Figure 5: Sema3E treatment reduces IgE and IgG1 synthesis after HDM re-exposure. Total **(A)** and HDM-specific IgE **(B)** as well as IgG1 **(C-D)** in the serum samples obtained from the chronically challenged mice treated with either Sema3E or saline was measured by ELISA upon HDM re-exposure (n=4 mice per group, *$P<0.05$ and **$P<0.01$).
